# Evolution of Leaf Morphoanatomical Characters in the *Catolesia* Clade (Asteraceae, Eupatorieae, Gyptidinae) Reveals the New Monotypic Genus *Nadia*

**DOI:** 10.3390/plants15121794

**Published:** 2026-06-10

**Authors:** Aryana Vasque Frota Guterres, Stéphani Karoline de Vasconcelos Bonifácio, Rafael Felipe de Almeida, Nádia Roque

**Affiliations:** 1 Departamento de Botânica, Instituto de Ciências Biológicas, Universidade Federal de Minas Gerais, Belo Horizonte 31270-901, Brazil; aryanavasque2008@hotmail.com (A.V.F.G.); stephanivasconcelos@gmail.com (S.K.d.V.B.); 2 School of Animal, Plant, and Environmental Sciences, University of the Witwatersrand, Johannesburg 2092, South Africa; 3 Instituto de Biologia, Universidade Federal da Bahia, Salvador 40170-115, Brazil

**Keywords:** campo rupestre, compositae, micromorphology, taxonomy, trichomes

## Abstract

The *Catolesia* clade (Asteraceae, Eupatorieae, Gyptidinae) comprises four genera (*Bahianthus*, *Catolesia*, *Lapidia*, and *Morithamnus*), mostly confined to the Espinhaço mountain range of Eastern Brazil. Although this lineage is statistically well supported in molecular phylogenetic studies, recent findings point to *Disynaphia praeficta* being currently placed in the *Catolesia* clade, making *Disynaphia* paraphyletic. We analysed, scored, and mapped 102 leaf anatomical characters from all species of the *Catolesia* clade and selected outgroups to test the placement of *D. praeficta* into this clade, proposing a new monotypic genus and a taxonomic synopsis for the *Catolesia* clade, besides standardising descriptive anatomical terminology. We recovered several homoplasies and synapomorphies circumscribing all lineages sampled in our study, including *Disynaphia* s.s. and the remaining sampled outgroups. Our results also corroborated the placement of *D. praeficta* within the Catolesia clade with high statistical support. The cuneate to truncate lamina base was recovered as a synapomorphy supporting the Catolesia clade, whereas a petiole with three vascular bundles, ducts distributed throughout the lamina, and collenchyma sheath cell extensions were recovered as synapomorphies supporting *Nadia praeficta* (B.L. Rob.) A.V.F. Guterres and R.F. Almeida as a new monospecific genus. We demonstrated how highly informative leaf morphoanatomical characters are for the systematics of Eupatorieae and Asteraceae, besides demonstrating that leaf morphoanatomical characters provide a robust phylogenetic signal for generic delimitation within Eupatorieae, even if characterised as homoplasies.

## 1. Introduction

The Eupatorieae Cass. is one of the largest tribes of Neotropical Asteraceae, comprising ca. 185 genera and 2462 species [1,2,3]. The monophyly of this tribe has been corroborated by several phylogenetic studies over the last two decades, placing it within the Heliantheae Alliance clade [4,5]. In the most comprehensive phylogenetic study of Eupatorieae to date, Rivera et al. [6] sampled 77 genera and ca. 160 species, mainly occurring in Brazil, revealing that most of the 13 subtribes proposed by King and Robinson [7] are not monophyletic. Gyptidinae R.M. King and H. Rob. is one of these subtribes, comprising ca. 30 genera mostly confined to savanna environments in eastern South America [7]. In recent years, this subtribe was the focus of studies re-evaluating its generic circumscriptions, resulting in the identification of new genera, such as *Catolesia* D.J.N. Hind, *Lapidia* Roque and S.C. Ferreira, and *Semiria* D.J.N. Hind [3,8,9].

The *Catolesia* clade is a small lineage mostly confined to the campos rupestres vegetation of Eastern Brazil, comprising four accepted genera [i.e., *Bahianthus* R.M. King and H. Rob. (1 sp.), *Catolesia* (3 spp.), *Lapidia* (1 sp.), and *Morithamnus* R.M. King, H. Rob. and G.M. Barroso (2 spp.)], alongside the recently placed *Disynaphia praeficta* (B.L. Rob.) R.M. King and H. Rob. as sister group, making *Disynaphia* paraphyletic, a genus currently composed of 16 species from South America [6]. This clade is vegetatively recognised by its shrub habit, with individuals varying from 2 to 4 m tall, with stem, leaves, and involucral bracts viscose (in *Bahianthus* and *Morithamnus*), and alternate to decussate, usually lax, leaves and a persistent, bristled pappus (except for *Catolesia*) [3]. Even though the *Catolesia* clade is well-supported by molecular phylogenetic data, the relationships within its genera are still unresolved [6], reinforcing the need to increase sampling.

Recent morphoanatomical studies in species of Asteraceae from campos rupestres reported that several abiotic conditions of this vegetation selected structural adaptations on macro (i.e., congested leaves) and micromorphological (i.e., diversity of trichome morphologies, hypoderm, sclereids, and crypts) leaf characters [10,11]. Additionally, morphoanatomical characters were recently revealed as informative to circumscribe taxonomic complexes [11,12,13]. However, convergent evolution (i.e., homoplasy) is recurrent in Asteraceae, reinforcing the importance of interpreting morphoanatomical traits within a phylogenetic framework [6].

Thus, the *Catolesia* clade is an excellent model group for studying the evolution of leaf morphoanatomical characters in Asteraceae, mainly to test their relevance to circumscribe all recently identified lineages of this clade. In particular, the phylogenetic placement and anatomical data of *Disynaphia praeficta* remain insufficiently investigated. The goal of this study was to describe leaf morphoanatomical characters from all species of the *Catolesia* clade and selected outgroups, to optimise them within a molecular phylogenetic framework, and to test for informative morphological characters (i.e., apomorphies and homoplasies) to circumscribe these lineages, including the placement of the intriguing *Disynaphia praeficta*. Additionally, we wanted to evaluate whether *Disynaphia praeficta* represents an independent lineage supported by diagnostic anatomical and phylogenetic characters.

## 2. Results

### 2.1. Morphology: Phyllotaxy and Shapes

**Figure 1:**
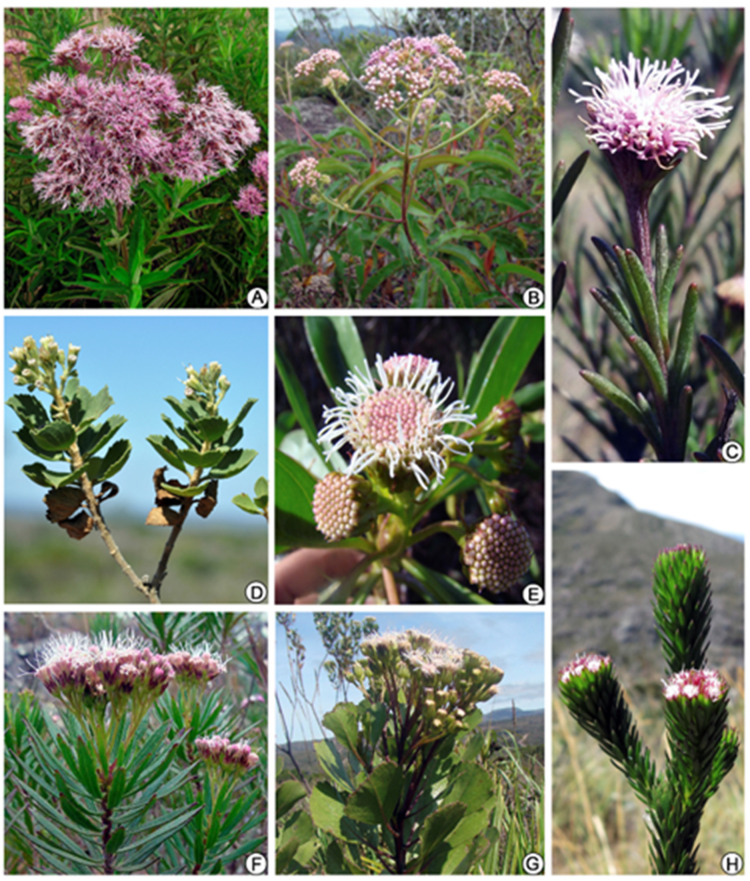
Field photographs of all lineages of the *Catolesia* clade and outgroups (**A**,**B**): (**A**) *Disynaphia multicrenulata*; (**B**) *Acritopappus confertus*; (**C**) *Catolesia monocephala*; (**D**) *Lapidia apicifolia*; (**E**) *Morithamnus crassus*; (**F**) *Disynaphia praeficta*; (**G**) *Bahianthus viscosus*; (**H**) *Catolesia mentiens.* Photographs (**A**) by L. Funez; (**B**,**E**–**G**) by N. Roque; (**C**,**H**) by V. Amorim; (**D**) by L. Barres.

A total of 102 structural characters (14 macro and 88 micromorphological) were scored and coded (Table 1) for both the Catolesia clade and outgroups (Figure 1). The sampled species shared densely leafy stems in the upper half and nodal regions detached after leaf senescence. Most species are viscid (on stems, leaves, and involucral bracts), except in *Lapidia* and *Catolesia*. The leaves’ posture is lax to congested, mostly alternate spiralled (except in *Morithamnus ganophyllus* and *Lapidia* with opposite and decussate leaves), varying from sessile to petiolate. The laminae are orbicular (*Lapidia*), spatulate (*Bahianthus*), aciculate (*Catolesia monocephala*), elliptic (*Morithamnus*), and linear (*Disynaphia praeficta*), commonly somewhat succulent or fleshy (except in *Bahianthus*), flat, naviculate (*Morithamnus*) or conduplicate (*Lapidia*), margins serrate or denticulate (*Bahianthus*, *Disynaphia praeficta* and *Lapidia*) and entire (*Morithamnus* and *Catolesia*).

### 2.2. Anatomy: Petiole

**Figure 2:**
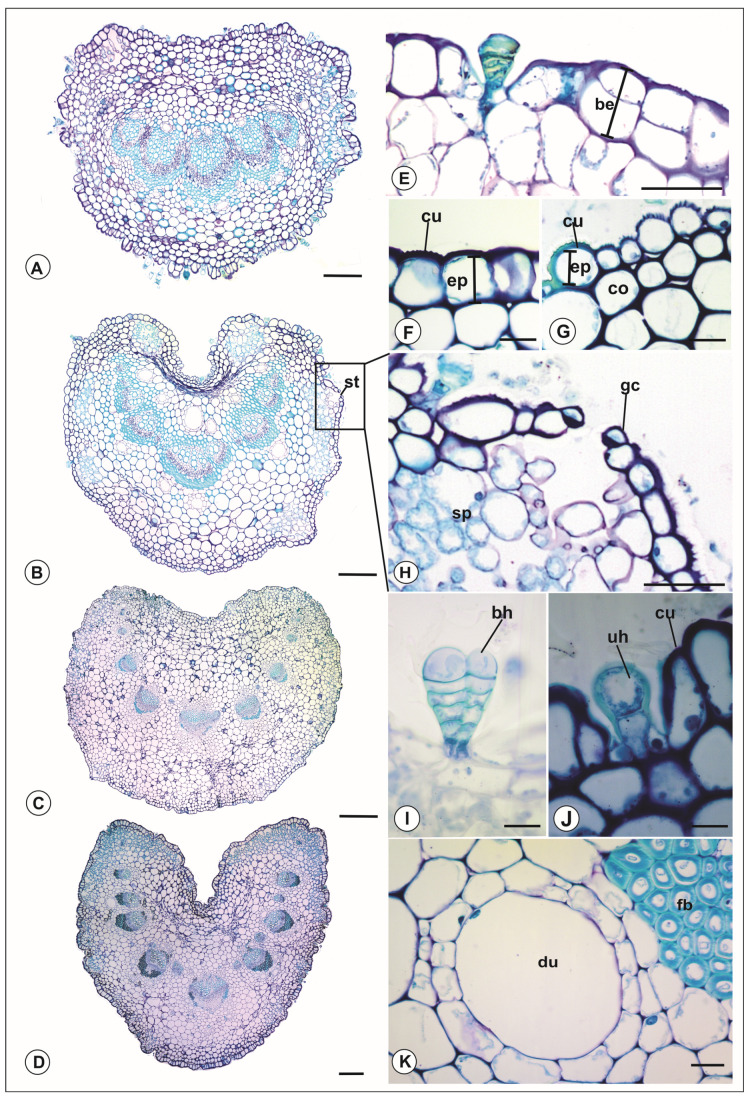
Petiole anatomy in transversal section: (**A**) Plane-convex contour of *Lapidia apicifolia*; (**B**) Concave-convex contour of *Bahianthus viscosus*; (**C**) Concave-convex contour of *Acritopappus confertus*; (**D**) Concave-convex contour in *Morithamnus ganophyllus.* (**E**) Inconspicuous biseriate epidermis in *Morithamnus crassus*; (**F**) Square-shaped epidermis of *Morithamnus crassus*; (**G**) Rounded epidermis of *Bahianthus viscosus*; (**H**) Raised stomata in *Bahianthus viscosus*; (**I**) Detail of a biseriate head of a trichome of *Lapidia apicifolia*; (**J**) Detail of a uniseriate head of a trichome of *Lapidia apicifolia*; (**K**) Midrib region showing fibres and ducts associated with the vascular bundles of *Lapidia apicifolia*. be = biseriate epidermis, bh = biseriate head, co = collenchyma, cu = cuticle, ep = epidermis, du = duct, fb = fibres, gc = guard cell, sp = spongy parenchyma, st = stomata, uh = uniseriate head. Scale bar = 50 µm in (**A**–**E**,**G**,**K**); 100 µm in (**F**,**H**); 20 µm in (**I**,**J**).

The contour varied from plane-convex in *Lapidia apicifolia* (Figure 2A) and concave-convex in *Bahianthus viscosus* and *Acritopappus confertus* (Figure 2B–D) in transversal sections. The epidermis is uniseriate in all studied species, except in *M. crassus*, which presents an inconspicuous biseriate epidermis with evidence of periclinal divisions in some cells (Figure 2E). The contour of epidermal cells is square-shaped in *Morithamnus crassus* (Figure 2F) in transversal section and rounded in *Bahianthus viscosus* (Figure 2G).

Most species frequently show undulations in the epidermal cell walls in the midrib (Figure 2A). Trichomes are predominantly glandular when present, usually with uniseriate heads in *Lapidia apicifolia* (Figure 2H,I) or biseriate heads in the remaining species (Figure 2I). The shape of the head of the trichome is globose with a uniseriate peduncle, inserted in depressions in *Lapidia apicifolia* (Figure 2I). The cuticle can be thick in *Morithamnus crassus* (Figure 2F) to thin in *Lapidia apicifolia* (Figure 2I) and striated in *Morithamnus crassus* and *Bahiathus viscosus* (Figure 2F–H) to smooth in *Morithamnus crassus* and *Lapidia apicifolia* (Figure 2E,I,J).

Another typical character is the slightly raised stomata in *Bahianthus viscosus* (Figure 2H) and spongy parenchyma (Figure 2H). Secretory ducts (Figure 2K) were observed in all species studied, including *L. apicifolia*. The conformation of vascular bundles is an open arch in all species, with a collateral unit in all species, showing a horizontal disposition (Figure 2A) and a U-shaped disposition (Figure 2B–D). Still, the number and arrangement of vascular bundles varied considerably among the analysed species, reaching up to 12 vascular bundles (Figure 2A–D) in the petiole region. Accessory bundles were observed in all species, except in *B. viscosus*. Xylem fibres associated with vascular bundles (Figure 2K) occurred in all petiolate species.

### 2.3. Anatomy: Leaf Blade

**Figure 3:**
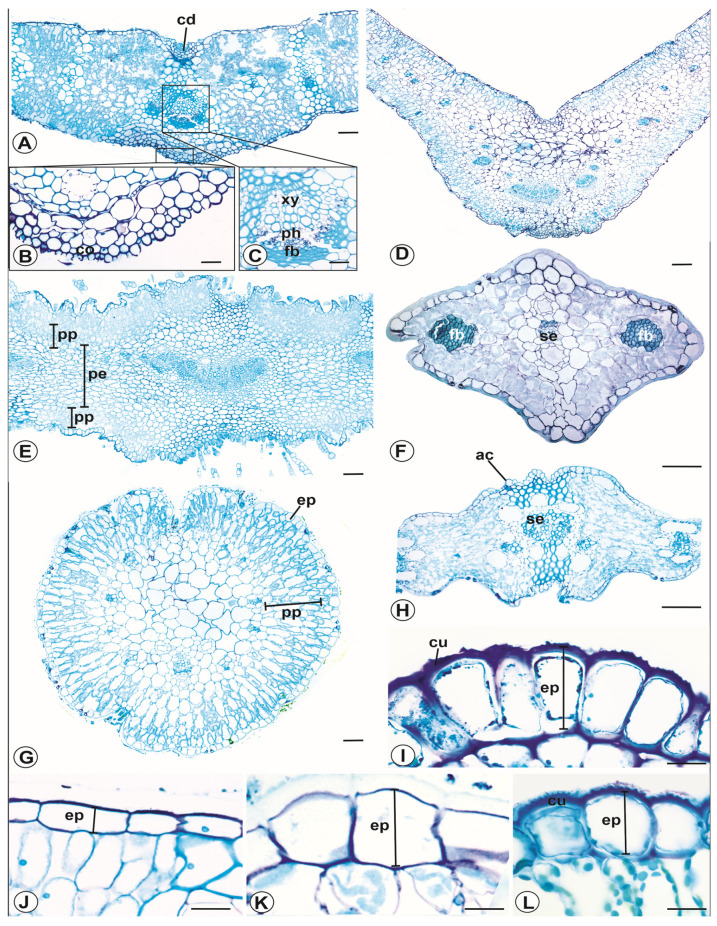
Lamina anatomy in transversal section: (**A**) Midrib plane-convex contour with central depression in *Bahianthus viscosus*; (**B**) collenchyma in *Bahianthus viscosus*; (**C**) fibres associated with vascular bundles in *Bahianthus viscosus*; (**D**) Midrib concave-convex contour in *Morithamnus ganophyllus*; (**E**) Midrib convex-convex contour in *Lapidia apicifolia*; (**F**) Parenchymatous bundles sheath extension in *Catolesia huperzioides*; (**G**) Midrib subunifacial contour in *Catolesia monocephala*; (**H**) Collenchymatous bundles sheath extension in *Disynaphia praeficta*; (**I**) Anticlinally elongated epidermis in *Morithamnus ganophyllus*; (**J**) Periclinally elongated epidermis in *Bahianthus viscosus*; (**K**) Squared-shaped contour of the epidermis in *Catolesia mentiens*; (**L**) Rounded-shaped contour of the epidermis in *Morithamnus ganophyllus*. ac = angular collenchyma, cd = central depression, co = collenchyma, cu = cuticle, ep = epidermis, fb = fibres, ph = phloem, pp = palisade parenchyma, pe = spongy parenchyma, se = sheath extension, xy = xylem. Scale = 100 µm in (**A**–**C**); 50 µm in (**D**–**L**).

The midrib contour of the studied species was plane-convex in *B. viscosus* (Figure 3A–C), concave-convex in *M. ganophyllus* (Figure 3D) and convex-convex in *C. huperzioides* (Figure 3E,F,H), except for *C. monocephala*, which showed subunifacial contour (Figure 3G). Some variation in the midrib contour was observed on the adaxial surface of the plane-convex contour in a central depression in *B. viscosus* (Figure 3A). In the midrib, the same proportion was observed between the mesophyll and semi-limb (Figure 3A,E) or the mesophyll was more voluminous than the semi-limb (Figure 3D,F,H).

The epidermis is uniseriate in all studied species with undulations and indentations on all or some parts of the epidermis, but mainly on the midrib in *L. apicifolia* (Figure 3E). Epidermal cell shape varied from anticlinally in *M. ganophyllus* (Figure 3I) to periclinally in *B. viscosus* (Figure 3J), elongated and squared in *C. mentiens* (Figure 3K), and rounded in *M. ganophyllus* (Figure 3L).

**Figure 4:**
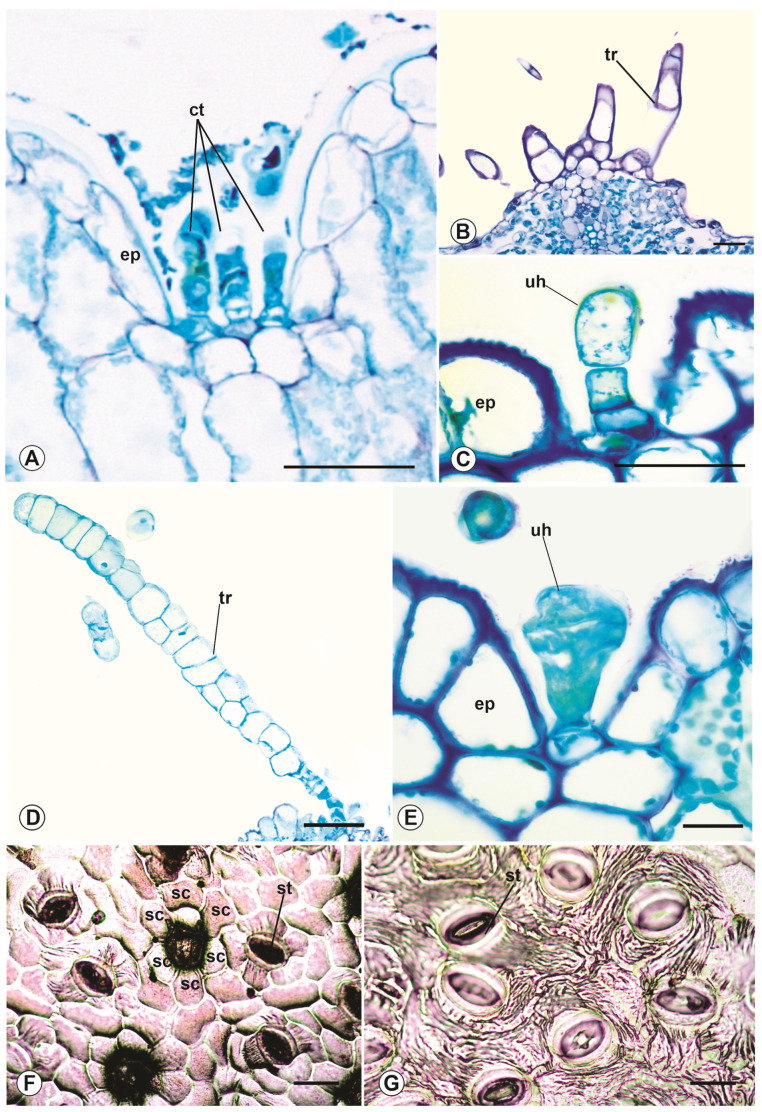
Trichome anatomy in transversal section (**A**–**E**) and paradermal view (**F**,**G**): (**A**) Cluster of trichomes in *Catolesia monocephala*; (**B**) Conical tector trichome in *Bejaranoa semistriata*; (**C**) Glandular trichome with globoid head in *Morithamnus ganophyllus*; (**D**) Biseriate glandular trichomes in *Lapidia apicifolia*; (**E**) Glandular trichomes with uniseriate heads in depressions in Morithamnus ganophyllus; (**F**) Actinocytic stomata in *Morithamnus crassus*; (**G**) Anomocytic stomata in *Disynaphia praeficta* with striated subsidiary cells. ct = cluster trichomes, sc = subsidiary cells, st = stomata, tr = trichome, uh = uniseriate head. Scale = 20 µm in (**A**); 50 µm in (**B**–**E**,**G**); 100 µm in (**F**).

Glandular trichomes (Figure 4A,C–E) were observed in all studied species, but they were absent in some analysed individuals of *Catolesia huperzioides*. Individual and paired (clustered) trichomes were also observed in *Catolesia monocephala* (Figure 4A). Tector trichomes were conical and not ramified in the outgroup species *Bejaranoa semistriata* (Figure 4B).

**Figure 5:**
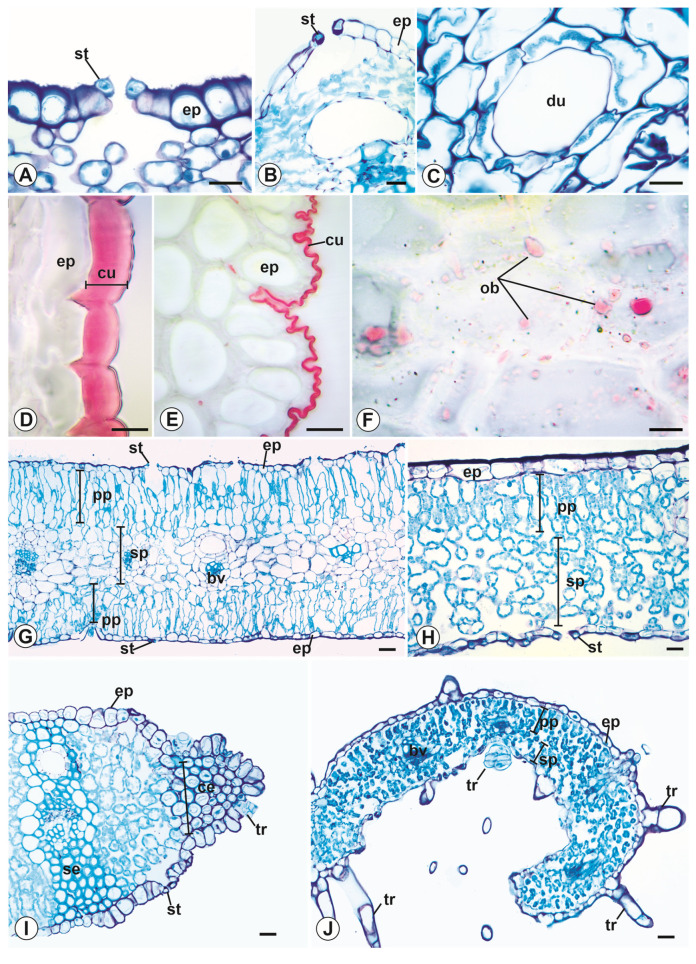
Anatomy of stomata, secretory structures, and semi-limb in transversal section. Material submitted to histochemical test, using Sudan IV for total lipids (**D**–**F**): (**A**) Plane stomata in *Acritopappus confertus*; (**B**) Raised stomata in *Disynaphia praeficta*; (**C**) Secretory ducts in *Acritopappus confertus*; (**D**) Thick cuticle in *Catolesia mentiens*; (**E**) Delgate and striate cuticle in *Acritopappus confertus*; (**F**) oil bodies in *Lapidia apicifolia*; (**G**) Isobilateral mesophyll in *Morithamnus crassus*; (**H**) Dorsiventral mesophyll in *Acritopappus confertus*; (**I**) Margin with sheath collenchymal cells extension in *Bahianthus viscosus*; (**J**) Revolute margin in *Bejaranoa semistriata*. bv = vascular bundle, ce = collenchymal cells extension, cu = cuticle, du = duct, ep = epidermis, fb = fibres, ob = oil body, pp = palisade parenchyma, se = bundles sheath extension, sp = spongy parenchyma, st = stomata, tr = trichome. Scale = 50 µm in (**A**,**I**,**J**); 100 µm in (**B**); 10 µm in (**C**–**F**); 200 µm in (**G**,**H**).

Stomata in the analysed species can be actinocytic in *Morithamnus crassus* (Figure 4F) or anomocytic in *Disynaphia praeficta* (Figure 4G), with insertion plane (Figure 5A) or raised (Figure 5B), such as in *Disynaphia praeficta* and *Lapidia apicifolia*. Ducts (Figure 5C) were found in all analysed species in the midrib, mesophyll and vascular bundles.

The cuticle might be thick in *C. mentiens* (Figure 5D) and thin in *A. confertus* (Figure 5E), the first being more commonly found and varying from striate in *A. confertus* (Figure 5E) to smooth in *C. mentiens* (Figure 5D). In addition, leaves can be amphistomatic in *Morithamnus crassus* (Figure 5G) and hypostomatic in *Acritopappus confertus* (Figure 5H).

Epidermal cell volume was constant, except in *Acritopappus confertus* and *Bejaranoa semistriata* (outgroups), where the adaxial surface of the epidermal cell was more voluminous (Figure 5H). The number of vascular bundles varied from 1 to 12, and bundle sheath extensions were parenchymatous in *Bahianthus viscosus* (Figure 5I) and *Catolesia huperzioides* (Figure 3F), but collenchymatous in *Disynaphia praeficta* (Figure 3H). Xylem fibres (Figure 3C,F) occurred in most species associated with the vascular bundles, mesophyll and margin. Regarding secondary metabolites, all species showed pectin-secreting walls and lipids (Figure 5D,E), highlighting oil bodies in the midrib and mesophyll in spongy parenchyma cells, including in *Lapidia apicifolia* (Figure 5F). The histochemical tests for detection using acid phloroglucinol-HCl for lignin and ferric chloride for phenolic compounds revealed a positive reaction in lignified cells in the vascular bundle and fibres in all studied species.

**Figure 6:**
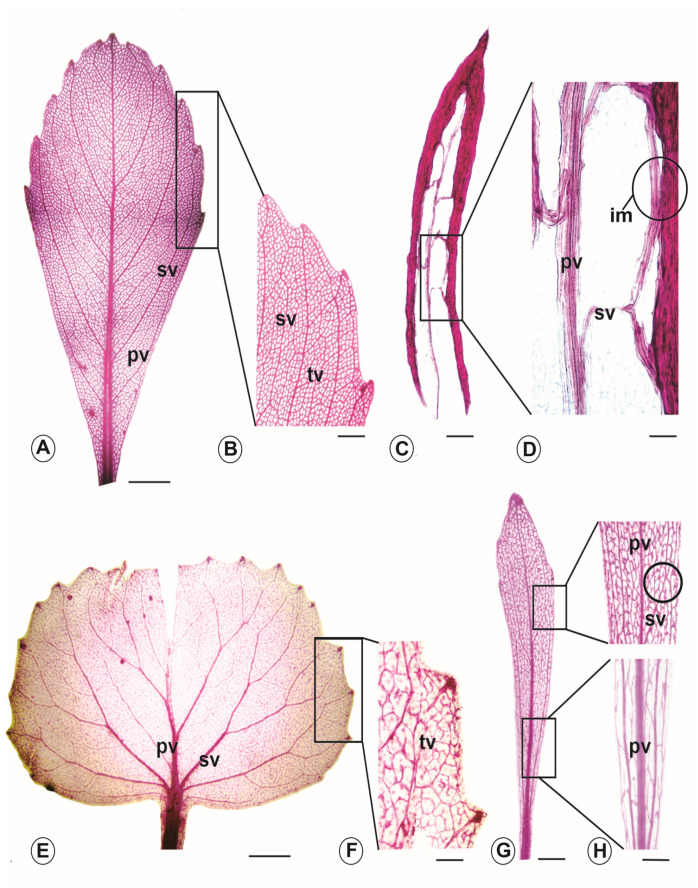
Leaf venation sections. (**A**–**D**) brochidodromous type in *Bahianthus viscosus* and *Catolesia huperzioides* with intramarginal veins (circle), respectively. (**E**,**F**) Actinodromous type in *Lapidia apicifolia*. (**G**,**H**) actinodromous type in *Disynaphia praeficta*. im = intramarginal veins, pv = primary veins, sv = secondary veins, tv = third veins. Scale = 1.4 cm in (**A**,**E**); 0.7 cm in (**B**–**D**,**F**–**H**).

The semi-limb showed isobilateral mesophyll (Figure 5G) as the most common, and dorsiventral (Figure 5H). The number of layers in the spongy parenchyma varied, and the cell shape varied from isodiametric in *Morithamnus crassus* (Figure 5G) to oval in *Acritopappus confertus* (Figure 5H). The leaf margin showed some uncommon characteristics, including sheath collenchymal cells extension (Figure 5I) in *Bahianthus viscosus* and revolute conformation in *B. semistriata* (Figure 5J). Venation patterns were essential to describe species varying from pinnate, brochidodromous (Figure 6A–D), and actinodromous venation was only found in *Lapidia apicifolia* (Figure 6E,F), and *Disynaphia praeficta* (Figure 6G,H). Intramarginal veins (Figure 6C,D) were uncommon only in *C. huperzioides*.

### 2.4. Phylogeny

**Figure 7:**
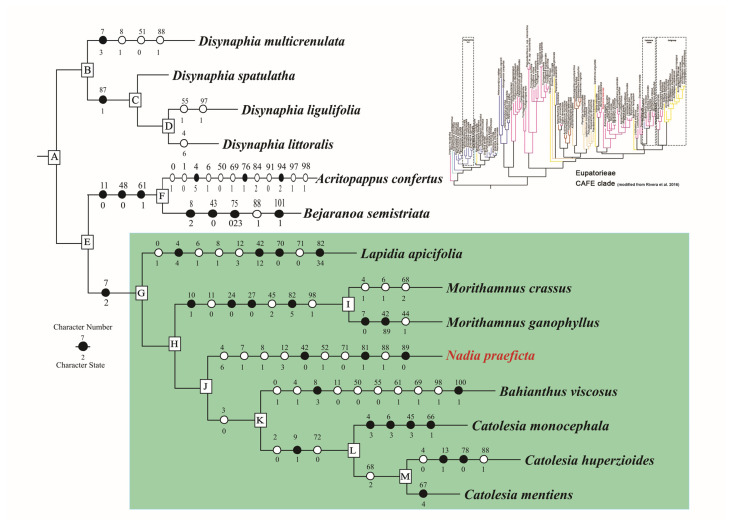
Character optimisation analysis of all 102 morphoanatomical characters on the Maximum Likelihood tree generated in this study and implemented on Mesquite software using the maximum likelihood criteria, and visualised on Winclada (ver. 1.0000) software. Black circles represent synapomorphies, and white circles represent homoplasies. A–M = clade names. Rivera et al. [6].

The combined plastid + nuclear dataset included 3809 analysed characters, of which 1055 characters were nuclear, and 2754 were from the chloroplast. Topologies produced by BI and ML analyses, separately based on nuclear and plastid datasets, did not exhibit incongruences, so we performed a combined analysis (Figure 7). Support values are presented on branches, with bootstrap values shown before posterior probabilities. The *Catolesia* clade was recovered as strongly monophyletic (100/1) and as a sister to a clade comprising the outgroups *Acritopappus confertus* + *Bejaranoa semistriata*. Combined plastid + nuclear datasets provided greater support for more clades than the results based on independent plastid or nuclear datasets. The BI and ML analyses recovered a fully resolved tree with 13 supported clades (Figure 7). All genera (*Lapidia*, *Morithamnus, Bahianthus*, and *Catolesia*) and *Disynaphia praeficta* within the *Catolesia* clade were recovered as monophyletic with support values as 100/1.

### 2.5. Character-Mapping

From all the 102 leaf morphoanatomical characters scored into our matrix (Table 2), clade C was recovered with one homoplasy (Character 87, state 1). *Acritopappus* was recovered with nine homoplasies (Characters 0, state 1; 1, state 0; 6, state 1; 50, state 0; 69, state 1; 84, state 2; 91, state 0; 97, state 1; and 98, state 1) and three synapomorphies (Characters 4, state 5; 76, state 1; and 94, state 2). *Bejaranoa* recovered with one homoplasy (Characters 88, state 1) and four synapomorphies (Characters 8, state 2; 43, state 0; 75, states 0, 2, 3; and 101, state 1) (Figure 7).

The *Catolesia* clade (Clade G) was recovered with one synapomorphy (Characters 7, state 2). *Lapidia* recovered with five homoplasies (Characters 0, state 1; 6, state 1; 8, state 1; 12, state 3; and 71, state 0) and four autapomorphies (Characters 4, state 4; 42, state 1,2; and 82, state 3,4). *Morithamnus* (Clade I) was recovered with three homoplasies (Characters 11, state 0; 45, state 2; and 98, state 1) and four synapomorphies (Characters 10, state 1; 24, state 0; 27, state 0; and 82, state 5).

*Disynaphia praeficta* was recovered with seven homoplasies (Characters 4, state 6; 7, state 1; 8, state 1; 12, state 3; 52, state 1; 71, state 0; and 88, state 1) and three autapomorphies (Characters 42, state 0; 81, state 1; and 89, state 0). *Bahianthus* recovered with eight homoplasies (Characters 0, state 1; 4, state 1; 11, state 0; 50, state 0; 55, state 0; 61, state 1; 69, state 1 and 98, state 1) and two autapomorphies (Characters 8, state 3 and 100, state 1). *Catolesia* (Clade L) was recovered with two homoplasies (Characters 2, state 0 and 72, state 0) and one synapomorphy (Characters 9, state 1). The remaining clades and species sampled in this study were recovered with at least a single homoplasy or apomorphy (Figure 7) but are not described here since the focus of this study relies only on generic circumscriptions related to the *Catolesia* clade.

### 2.6. Taxonomic Treatment

#### 2.6.1. Catolesia Clade

**Figure 8:**
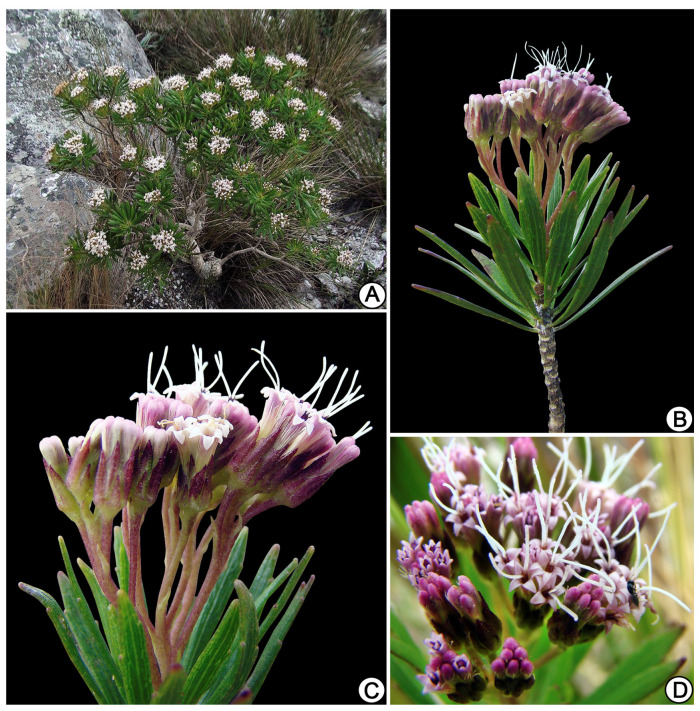
*Nadia praeficta* (B.L. Rob.) A.V.F. Guterres and R.F. Almeida: (**A**) Shrub between rock cleft in campo rupestre vegetation; (**B**) Synflorescence corymbiform with leaves arranged at the apex of the branch; (**C**) Involucral bracts in four series; (**D**) Capitula with 5–6 flowers. Photographs (**A**) by Guarçoni, E.; (**B**–**D**) by Roque, N.).

**Figure 9:**
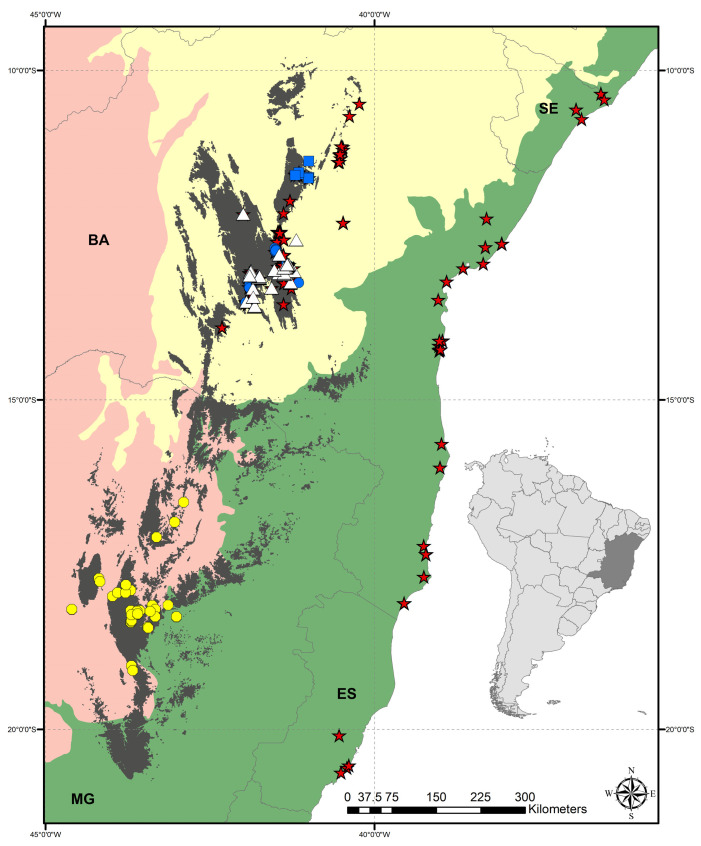
Distribution map of all genera of the *Catolesia* clade. Yellow circle—*Nadia*, Red star—*Bahianthus*, White triangle—*Catolesia*, Blue square—*Lapidia*, Blue circle—*Morithamnus*. Green—Atlantic Forest biome, Pink—Cerrado biome, and Yellow—Caatinga biome.

**Diagnosis.** Shrubs, candelabra-shaped habit, densely leafy in the upper half and nodal region detached after the senescent leaves, most glabrous and viscid (except in *Lapidia* and *Catolesia*), leaf blades with convex-convex contour in the midrib and mesophyll isobilateral, floral receptacle glabrous, pappus bristled (except in *Catolesia*, which is absent to shortly coroniform).
**Notes.** The *Catolesia* clade (Figure 1C–H and Figure 8) includes five monophyletic genera and eight species primarily endemic to campos rupestres within the Espinhaço mountain range in the States of Bahia and Minas Gerais, Eastern Brazil (Figure 9). *Bahianthus viscosus* is the only species within this clade with a broader distribution range outside campos rupestres, also commonly occurring in the restingas of the States of Bahia, Espírito Santo, and Sergipe, Brazil (Figure 9). An identification key to all genera within this clade is presented below.

#### 2.6.2. Key to the Genera of the Catolesia Clade

1. Lamina aciculate to subulate, succulent; receptacle paleaceous; pappus absent or defective (a short dentate to laciniate crown ca. 0.1 mm long) ……………………… *Catolesia*
- Laminas orbicular, obovate, linear to narrowly elliptic, semi-succulent or not; receptacle epaleaceous; pappus with 20–30 persistent shortly ciliate-dentate bristles in one series …………………………………………………………………………………………………… 2
2. Leaves 8–15 cm long, naviculate, margins entire; capitulescence persistent with age, persistent and becoming overtopped by sub-floral innovation; involucral bracts ca. 35; flowers 25–100 ……………………………………………………………………… *Morithamnus*
- Leaves 1.8–7.0 cm long, flat to conduplicate, margins serrate to dentate; capitulescence not persistent with age; involucral bracts 10–20; flowers 5–22…………………………… 3
3. Shrubs tomentose, without viscosity; leaves opposite, decussate; laminas orbicular, conduplicate ………………………………………………………………………………… *Lapidia*
- Shrubs glabrous, viscid; leaves alternate; laminas linear to narrow elliptic, obovate, flat ……………………………………………………………………………………………………… 4
4. Lamina coriaceous, oblanceolate, 1.3–2.6 cm wide, petiolate (petiole 1.5–2.5 cm long), involucral bracts 18–20; flowers 15–22 ………………………………………………*Bahianthus*
- Lamina somewhat fleshy, linear or narrowly elliptic, 0.1–0.6 cm wide, sessile to petiolate (petiole 2–4 mm long); involucral bracts 10–12; flowers 5–6 …………………… *Nadia*

#### 2.6.3. *Bahianthus* R.M. King and H. Rob. [14]

**Type species**. *Bahianthus viscosus* (Spreng.) R.M. King and H. Rob.
**Diagnosis.** Shrubs laxly ramified, viscid; leaves alternate, densely spirally inserted, petiole 1.5–2.5 cm long, leaf blades oblanceolate, serrate; capitulescence corymbiform, lax; receptacle flat, epaleaceous; flowers 15–22; cypsela 4–5-costate, pappus ca. 30-bristled.
**Comments**. *Bahianthus* comprises a single species, *Bahianthus viscosus.*

##### 2.6.3.1. *Bahianthus viscosus* (Spreng.) R.M. King and H. Rob. [14] (Figure 1G)

**Basionym**. *Mikania viscosa* Spreng., Neue Entdeck. Pflanzenk. 1: 277 (1820).
**Type.** BRAZIL—Bahia • Nazaré; F. Sellow 218; lectotype, (designated here): K000017345!; isotype: P00708646!).
**Distribution, habitat, and ecology**. The original material of *Mikania viscosa* was collected by Sellow in 1820 in the municipality of Nazaré, State of Bahia, Brazil. According to the TL-II, Sellow deposited his type specimens at the B herbarium with duplicates sent to several European herbaria. Since specimens of Asteraceae were lost at B during the bombing event in WWII, we assume that the holotype of *M. viscosa* is lost and designate the isotype housed at K as lectotype.

##### 2.6.3.2. *Catolesia* D.J.N. Hind [9]

**Diagnosis**. Shrubs imbricate, densely ramified; leaves spirally alternate, sessile, leaf blades aciculate, fleshy; capitulescence corymbiform, congested and surrounded by leaves or capitulum solitary at the apex; receptacle conic to convex, paleaceous; flowers (10) 60–100; cypsela 5-costate; pappus absent to a short dentate to laciniate crown ca. 0.1 mm long.
**Distribution, habitat, and ecology.** *Catolesia* comprises three species described in the last two decades, all endemic to the Diamantina Plateau in the State of Bahia, Brazil. The genus presents several distinct characters when compared to the other genera in the clade, such as leaves mostly strongly imbricate (vs. lax or congest), sessile (vs. most petiolate), laminae aciculate or subulate, succulent, receptacle paleaceous (vs. receptacle epaleaceous) and pappus absent or defective (vs. pappus of bristles).

###### 2.6.3.2.1. *Catolesia huperzioides* Roque, H. Rob. and A.A. Conc. [15]

**Type**. BRAZIL—Bahia • Mucugê: Chapada Diamantina, Serra do Esbarrancado, 12°43′ S, 41°30′ W, 5 Sep. 2006; fl.; A.A. Conceição and P.D. Carvalho 1804; holotype: HUEFS112499!; isotypes: ALCB!, [K000768729!, IBGE00063945!).

###### 2.6.3.2.2. *Catolesia mentiens* D.J.N. Hind [9] (Figure 1H)

**Type**. BRAZIL–Bahia • Abaira: Serra das Brenhas, 1860 m, 13°19′ S 41°52′ W, 22; Oct. 1992, W. Ganev 1313; holotype: [HUEFS12377!]; isotypes: [K000054551!, SPF00088148!, US00901668!].

###### 2.6.3.2.3. *Catolesia monocephala* Roque and S.C. Ferreira [16] (Figure 1C)

**Type**. BRAZIL–Bahia • Palmeiras: Serra do Esbarrancado; 12°44′39.7″ S, 41°30′24.9″ W; 1421 m, 29 Sep. 2015; V.O. Amorim 408; holotype: [ALCB054090!]; isotypes: [HUEFS000100244808!, SPF00240700!, US!].

##### 2.6.3.3. *Lapidia* Roque and S.C.Ferreira [3] (Figure 1D)

**Type species**. *Lapidia apicifolia* Roque and S.C. Ferreira.
**Diagnosis**. Shrubs laxly ramified; leaves opposite-decussate, petiolate, blades orbicular to obovate, conduplicate; capitulescence corymbiform, lax; receptacle flat to slightly convex, epaleaceous, flowers 18–20; cypsela 5-costate, pappus 20–25-bristled.
**Comments**. *Lapidia* is a recently described monotypic genus endemic to the Chapada Diamantina region in the State of Bahia, Brazil. *Lapidia apicifolia* is recognised by a set of characters including the presence of tomentose indumentum, without viscidity, leaves opposite, decussate and laminas orbicular to obovate, conduplicate, and short petiolate (4–6 mm long); receptacle epaleaceous; flowers 18–20; cypsela 5-ribbed, pappus of 20–25-bristled.

###### *Lapidia apicifolia* Roque and S.C. Ferreira [3] (Figure 1D)

**Type**. BRAZIL–Bahia • Morro do Chapéu, Cachoeira do Ferro Doido, 11°37′40.5″ S, 41°00′1.4″ W, 900 m alt., 16 September 2015, M.G. Staudt and L. Barres 74 (holotype: ALCB036655!; isotypes: HUEFS000279445!, RB01297983!, RB01297987!, SPF00226300!, US01269483!).

##### 2.6.3.4. *Morithamnus* R.M. King, H. Rob. and G.M. Barroso [17] (Figure 1E)

**Type species**. *Morithamnus crassus* R.M. King, H. Rob. and G.M. Barroso.
**Diagnosis**. Shrubs laxly ramified, viscid; leaves alternate to opposite, decussate, petiolate, blade obovate to oblanceolate, naviculate; capitulescence corymbiform, lax; receptacle flat to slightly convex, epaleaceous; flowers 25–100; cypsela 4-costate, pappus 20–25-bristled.
**Distribution, habitat, and ecology.** *Morithamnus* comprises two species endemic to the Chapada Diamantina, State of Bahia, Brazil.

###### 2.6.3.4.1. *Morithamnus crassus* R.M. King, H. Rob. and G.M. Barroso [17] (Figure 1E)

**Type**. BRAZIL–Bahia • Mucugê estrada que liga Mucugê com Andaraí a 11 km, 1150 m alt.; 27 July 1979; R.M. King, S.A. Mori, T.S. dos Santos and J.L. Hage 8166; holotype: [RB00287798!]; isotypes: [BM001009254!, CEPEC18271!, G00300912! M-0029556!, US2855718!, US 2855719!, W1981-0002328!].

###### 2.6.3.4.2. *Morithamnus ganophyllus* (Mattf.) R.M. King and H. Rob., Phytologia 46: 300. 1980. Basionym: *Eupatorium ganophyllum* Mattf., Notizbl. Bot. Gart. Berlin-Dahlem 9: 379

**Type**. BRAZIL—Bahia • Rio de Contas: Bom Jesus, Jul. 1913; P. von Luetzelburg 308; holotype: [M0009526!; isotype: B†?, IAN73527!, US2902332!].

##### 2.6.3.5. Nadia A.V.F. Guterres and R.F. Almeida, Gen. Nov. (Figure 1D and Figure 8A–D)

**Basionym**. *Eupatorium praefictum* B.L. Rob.
**Type species**. *Nadia praeficta* (B.L. Rob.) A.V. F.Guterres and R.F. Almeida, comb. nov.
**Diagnosis**. Shrubs, branches viscid. Leaves spirally alternate, sessile to petiolate, leaf blade linear to narrowly elliptic, glabrous, viscid. Capitulescence corymbiform, terminal, heads pedunculate; involucre subimbricate, 3-seriate; receptacle flat, epaleaceous. Flowers 5–6 per head, corolla pink to white, anther apical appendage obtuse, base sagittate, style branches cylindric, elongate, slightly swollen, base slightly enlarged, glabrous. Cypsela prismatic, 5-costate, ribs bristled, phytomelanin present, carpopodium asymmetric, pappus bristled, uniseriate, stray, bristles 18–20, persistent, unequal.

###### *Nadia praeficta* (B.L.Rob.) A.V.F. Guterres and R.F. Almeida, Comb. Nov.

**Type**. BRAZIL–Minas Gerais • Voyage d’Auguste de Saint-Hilaire from 1816 to 1821, A. Saint-Hilaire Catalogue B 1990; holotype: [P00603167!; isotype: GH00257141!, GH00007926!, P03781680!].
**Description**. Shrubs, candelabra-shaped, 0.6–1.5 m tall, branches sympodial, brown, puberulous, glabrescent, viscid. Leaves spirally alternate, congested at the apex of branches, nodal region detached after the falling leaves, sessile to petiolate, petiole 2–4 mm long, glabrescent; leaf blade 2–5 × 0.1–0.6 cm, linear, slightly discolour, apex acute, callose, margin dentate, base attenuate, glabrous on both surface, venation actinodromous, viscid, glandular trichomes uniseriate head, on both surfaces. Capitulescence corymbiform, terminal; heads discoid, homogamous, pedunculate, peduncle ca. 1 cm long, vinaceous, receptacle flat, epaleaceous. Involucre campanulate, 0.7–1 × 0.2–0.3 cm; involucral bracts 8–10, sub-imbricate, 3-seriate, unequal, the outer bracts obovate to lanceolate, 3.5–5 × 1.5–2 mm, apex acute, margin entire, base attenuate, the inner bracts obovate, 1.5–2 × 0.5–1 mm, apex acute, margins lacerate, base truncate, glabrous, persistent. Flowers 5–6 per head, bisexual, corolla tubulose 6–7 × ca. 1 mm, pink to whitish, corolla-lobes triangular glabrous, anthers 3–4 mm long, vinaceous, apical anther appendage obtuse, wider than longer, base sagittate, style white, ca. 8 mm long, style branches 4–6 mm long, cylindrical at base, glabrous. Cypsela 2.9–3 × 0.5–1 mm, prismatic, 5-costate, ribs bristled, blackish, carpopodium asymmetrical, pappus bristled, uniseriate, 4.3–5.5 mm long, stray, bristles 18–20, persistent, unequal.
**Additional material examined**. Brazil. Minas Gerais. Botumirim: Parque Estadual de Botumirim, 16°51′12″ S, 43°02′12″ W, 13 October 2019, P.A. Junqueira et al. 382 (BHCB); Diamantina, Guinda, Adjacente à estrada Conselheiro Mata, 18°15′47″ S, 43°41′38″ W, 1446 m alt., 15 November 2010, N.F.O. Mota et al. 1801 (BHCB); Diamantina, Parque Nacional das Sempre Vivas, 17°53′27″ S, 43°42′17″ W, 1176 m alt., 4 September 2019, A.E. Brina s.n. (BHCB 203491); Itacambira: Serra do Resplandecente, 17°4′59.61″ S, 43°18′43.27″ W, 1306 m alt., 28 October 2009, E.K.O. Hattori et al. 939 (BHCB); Joaquim Felício: Parque Estadual da Serra do Cabral- Estrada Joaquim Felício para Várzea da Palma, 17°42′29.81″ S, 44°11′22.69″ W, 1002 m alt., 3 November 2009, E.K.O. Hattori and J.A.N. Batista 1043 (BHCB); Rio Vermelho: Pedra menina, 11°35′29″ S, 41°12′28″ W, 1460 m alt., 9 September 1986, T.B. Cavalcanti et al. s.n. (BHCB 83960); Rio Vermelho, Serra da Pedra Menina- Distrito de Pedra Menina, 18°06′57″ S, 43°08′16″ W, 1530 m alt., 26 August 2008, N.F.O. Mota and et al. 1372 (BHCB); São Gonçalo do Rio Preto: Parque Estadual do Rio Preto, 18°07′14″ S, 43°22′43″ W, 1113 m alt., 24 September 2019, P.B. Meyer and P.A. Junqueira 3591 (BHCB).
**Distribution, habitat, and ecology.** *Nadia* is a monotypic genus endemic to the campos rupestres from the Espinhaço mountain range, State of Minas Gerais, Brazil (Figure 9).
**Etymology**. The genus honours Dr Nadia Roque (1970–2024), Asteraceae specialist, friend and mentor, due to her enormous contribution to studies in Neotropical Asteraceae, mainly with the tribes Barnadesieae, Eupatorieae, Heliantheae, and Mutisieae. She also published the first books and book chapters on Asteraceae morphology in the Neotropics, which have become important references for new students working with Asteraceae taxonomy in the Neotropics.
**Preliminary IUCN conservation assessment.** *Nadia* shows an Extent of Occurrence (EOO) of 25,286.792 km^2^ and an Area of Occupancy (AOO) of 13,143.006 km^2^, with the AOO based on an auto-value cell width of 30 km, being classified as Least Concern (LC).

## 3. Discussion

Leaf anatomy has constantly been used in circumscribing taxonomic ranks, gaining ground in evolutionary studies [18,19,20,21]. Recovering one synapomorphy (cuneate to truncate foliar base) circumscribing the *Catolesia* clade, including *Disynaphia praeficta*, was a notable aid to the taxonomy of this group and corroborated Rivera et al. [6] previous phylogenetic study.

### 3.1. Petiole

The anatomy of petioles has been a poorly explored character (number 30 in Table 1) in systematic studies of Asteraceae, with few studies highlighting its relevance or demonstrating that it is under-sampled and in need of additional descriptions [22]. Nonetheless, recent studies have shed some needed light on the relevance of petiole anatomy studies in Asteraceae [5,22]. Characters related to the surface of the petiole contour have been used in the identification at the species level, as well as vascular arrangement, primarily based on the character states related to the number of bundles, arch conformation, size differences, and accessory bundles [5,22]. The glandular trichome ellipsoid head (character 24, state 0) and glandular trichome insertion (character 27, state 0) were relevant and recovered as a synapomorphy for clade I. Still, the vascular bundle number (character 42, state 8 and 9) was recovered as a synapomorphy, and the vascular bundle arrangement (character 44, state 1) was recovered as a homoplasy for *M. ganophyllus*. Finally, petioles with three vascular bundles (character 42, state 0) were recovered as an autapomorphy supporting the recognition of *Nadia praeficta* as a new monotypic genus. The taxonomic relevance of these anatomical characters might be evidenced by the conformation of vascular bundles in open arches, being one of the most conserved characters in the angiosperm classification [22]. In the present study, our data indicate the relevance of anatomical character optimisation for the systematics of Eupatorieae [2].

### 3.2. Leaf Blade

The leaf blade had the highest number (56) of relevant characters (i.e., homoplasies and synapomorphies) for the *Catolesia* clade, being also the case for other groups of flowering plants [23,24]. Among the general leaf anatomical characters, the contour is frequently used in descriptions, besides the cells and tissues of the midrib [21,22]. In the *Catolesia* clade, the midrib unifacial contour in *Catolesia monocephala* was the most distinct in the clade, being recovered as an autapomorphy for this species. Unifacial leaves (character 45, state 3) are not typical in Asteraceae, being only recorded in *Senecio* L. (Senecioneae), indicating a reduction in the adaxial surface, which is related to acicular and succulent leaves, occurring in the development of the leaf primordium [25]. Still, the absence of chlorophyll parenchyma in the midrib (Character 78, state 0) was recovered as a synapomorphy for *C. huperzioides*. The dermal system showed a region with the most significant number of informative characters in the *Catolesia* clade, especially those related to the epidermis and trichomes [18,26,27,28]. Previous studies in Asteraceae have also presented evolutionary interpretations for epidermal characters. Lusa et al. [21] recovered the biseriate epidermis as a possible synapomorphy for *Paralychnophora* MacLeish (Vernonieae), corroborating our results, only for *M. crassus* (Figure 2F). Additional studies focusing on Asteraceae have also corroborated the relevance of epidermal anatomical characters to the systematics of this family [11,21,23].

Trichomes are Asteraceae’s most informative morphoanatomical character, varying in type, shape, cell number, insertion, and distribution in the epidermis [26]. The anatomy and morphology of trichomes have been widely explored in systematic studies in Asteraceae, being conserved across different taxonomic ranks [29,30]. In the *Catolesia* clade, glandular trichomes were the predominant secretory structures and were important in the ancestral character reconstruction [19,31]. Trichomes in clusters (Character 66, state 1) were recovered as a synapomorphy for *C. monocephala*. In addition, trichome distribution (Character 67, state 4) was recovered as a synapomorphy for the *C. mentiens*. The chemical composition of these trichomes is a mixture of phenolics, pectin and lipids, which were also recovered as homoplasies in our analyses. Even though this character is homoplastic within the *Catolesia* clade, it is still relevant to species identification [32], functional mechanisms, and defence processes in Asteraceae [26]. Tector trichomes were scarce in the analysed species, but they are also widely used in taxonomic and evolutionary studies in Asteraceae [18,27]. In our results, conical tector trichomes were exclusive to species of *Disynaphia* s.s., easily differentiating them from *Disynaphia praeficta* and the remaining members of the Catolesia clade.

Secretory ducts have also been used in several taxonomic studies in Asteraceae [26,32], being also present in the Catolesia clade and the distribution for all lamina as a synapomorphic character (Character 81, state 1) for *Disynaphia praeficta*. The stomata were also an informative character in the Catolesia clade since the most common type in Asteraceae is the anomocytic, with the anisocytic being rarely recorded [31,33]. The actinocytic stomata (Character 68, state 2) were an essential homoplasy for *M. crassus*. The mesophyll dorsiventral in the semi-limb (Character 91, state 0) is the most cited tissue in anatomical descriptions of Asteraceae leaves [11,21], recovered as a homoplasy for *A. confertus*.

Few studies were found in the literature regarding venation patterns in Asteraceae. Nonetheless, some characters were informative in morphoanatomical analyses, such as general venation pattern, with the brochidodromous type being the most reported for species of Asteraceae [5,33,34,35]. This character was important for *L. apicifolia* and *D. praeficta*, which presented the actinodromous venation pattern (Character 12, state 3) as a homoplasy for these genera. Additionally, venation patterns have been used to segregate the new genus *Vickia* (Gochnatieae) [36].

### 3.3. Catolesia Systematics and Disynaphia praeficta

*Disynaphia praeficta* is currently placed in Disynaphiineae, a subtribe comprising six genera [2]. *Disynaphia* encompasses 16 South American species, with 12 of them occurring in Brazil and eight endemics to this country. The recent placement of *Disynaphia praeficta* within the evolutionary distantly related *Catolesia* clade is not a surprise when considering King and Robinson’s [7] classical studies. These authors proposed the combination of the name *Eupatorium praefictum* B.L. Rob. into *Disynaphia* based on the five-flowered capitulum since this species was glabrous and did not show the base of the anthers hastate, all characters common to *Disynaphia* s.s. Thus, based on molecular phylogenetic studies, Rivera et al. [6] recovered *Disynaphia praeficta* within the *Catolesia* clade and suggested that this name should be combined out of *Disynaphia* to render the subtribe Disynaphiineae monophyletic. Our results corroborate these authors’ findings, reinforcing that *Disynaphia praeficta* is an independent lineage placed within the *Catolesia* clade, sister to the *Bahianthus* + *Catolesia* clade. Recently, Silva et al. [37] analysed the cypsela morphoanatomy of all six genera of Disynaphiineae and stated that *Disynaphia praeficta* is the only species within this subtribe with very distinctive characters, such as asymmetrical carpopodium and secretory ducts in the pericarp, further corroborating our results.

Curiously, the *Catolesia* clade comprises five genera and eight species that occur mainly in campos rupestres, sharing many morphological and anatomical characters selected for this habitat. The recognition of a new monotypic genus, *Nadia* (*N. praeficta*), in a small clade with a restricted distribution is not unusual in Asteraceae [3,38]. According to different authors [39] this is a common pattern in Brazilian Asteraceae, with one larger and more widespread genus sister to two or three smaller and isolated genera endemic to campos rupestres. In this type of vegetation, ecological traits are likely directed toward adaptive convergence, as also evidenced in the subtribe Lychnophorinae [21]. This way, it can reflect similar morpho-ecological adaptations resulting in a high taxonomic diversity and endemism level in this environment. These patterns further suggest that the leaf anatomical traits observed in the *Catolesia* clade may reflect a combination of environmental filtering and convergent evolution associated with xeromorphic conditions.

## 4. Materials and Methods

### 4.1. Sampling

Leaf samples were obtained from field collections performed from 2018 to 2019 and/or herbarium sheets (ALCB, BHCB, and HUEFS; acronyms according to Thiers [40]. Completely expanded leaves from apical stems of 3–5 specimens of different populations, whenever possible, were selected for fixation in FAA 50 (formaldehyde-acetic acid-50% ethyl alcohol, 1:1:18 by volume) and immediately submitted to vacuum. All field-collected samples remained immersed in FAA 50 for 48 h and then preserved in 50% ethanol. Vouchers were collected and herborized according to [41] and deposited at the ALCB and BHCB herbaria (Table 3). The herborized samples were submitted to the herborization reversion method following Smith and Smith [42,43].

We sampled all eight species currently placed in the *Catolesia* clade [*Bahianthus viscosus* (Spreng.) R.M. King and H. Rob., *Catolesia huperzioides* Roque, H. Rob. and A.A. Conc., *Catolesia mentiens* D.J.N. Hind, *Catolesia monocephala* Roque and S.C. Ferreira, *Disynaphia praeficta* (B.L. Rob.) R.M. King and H. Rob., *Lapidia apicifolia* Roque and S.C. Ferreira, *Morithamnus crassus* R.M. King, H. Rob. and G.M. Barroso and *Morithamnus ganophyllus* (Mattf.) R.M. King and H. Rob.] and *Acritopappus confertus* (Gardner) R.M. King and H. Rob. (Figure 1B) and *Bejaranoa semistriata* (Baker) R.M. King and H. Rob. as outgroups according to [6]. Additionally, we sampled four species of *Disynaphia* s.s. [*Disynaphia ligulifolia* (Hook. and Arn.) R.M. King and H. Rob., *Disynaphia littoralis* (Cabrera) R.M. King and H. Rob., *Disynaphia spathulata* (Hook. and Arn.) R.M. King and H. Rob., and *Disynaphia multicrenulata* (Baker) R.M. King and H. Rob. (Figure 1A)] as the root of our analyses to aid in differentiating and circumscribing *D. praeficta*. Species sampled from the *Catolesia* clade, and the outgroups are found in Table 3.

### 4.2. Anatomy

Both field and herborized specimens preserved in 50% ethanol were then dehydrated in an ethyl alcohol series. The resin laminae were mounted from transversal and longitudinal sections of 6–10 µm thickness, obtained from (2-hydroxyethyl)-methacrylate embedded portions [44]. The distal third region of the petiole, midrib, semi-limb, margin, apex, and base of the lamina were sectioned in a Zeiss Hyrax M40 (Zeiss, Walldorf, Germany) microtome, posteriorly stained with 0.05% toluidine blue in acetate buffer pH 4.7 [45] and mounted in Entellan^®^. The laminae were analysed and photographed using an Olympus CX41 microscope coupled with an LC20 camera and subsequently arranged into plates using the CorelDRAW 2020^®^ software. All laminae produced and mounted are stored at PlantSeR—Plant Secretion and Reproduction Lab in Universidade Federal de Minas Gerais (UFMG). Additional leaf blade material from all sampled species was also diaphanized to analyse venation patterns. Samples were immersed in 5% sodium hydroxide for 24 h, washed in distilled water, and stained with a solution of 1% basic fuchsin diluted in 95% ethyl alcohol [46], and mounted in glycerine-gelatine [47]. All venation pattern descriptions followed [48]. The images were obtained with a Canon PowerShot A650 camera (Canon Inc., Tokyo, Japan) coupled with a Zeiss stereomicroscope. The identification of classes of secondary metabolites was performed in the resin laminae using ruthenium red for pectates and mucilage [49], ferric chloride for phenolic compounds and Sudan IV for total lipids [50] and acid phloroglucinol-HCl for structural phenolic compounds (lignin) [50].

### 4.3. Phylogeny

Sequences from two plastid (*ndhI*, *ndhF*) and two nuclear (External and Internal Transcribed Spacers) regions were retrieved from GenBank (Table 4), aligned using Muscle [51] implemented on Geneious software v.4.8 [52], with subsequent adjustments in the preliminary matrices made manually. All trees were rooted in all the species sampled of *Disynaphia* s.s., one of the first lineages to arise in Eupatorieae, according to [6]. A combined phylogenetic analysis of plastid + nuclear regions was performed using the Bayesian inference and Maximum Likelihood criteria. We selected the molecular model using hierarchical likelihood ratio tests (HLRT) on JModelTest 2 software [53]. Both model-based methods were conducted with a mixed model (GTR+G+I) and unlinked parameters, using MrBayes 3.1.2 [54] and raxmlGUI2 [55]. For the Bayesian inference, the Markov Chain Monte Carlo (MCMC) was run using two simultaneous independent runs with four chains each (one cold and three heated), saving one tree every 1000 generations for ten million generations. We excluded 25% of retained trees as ‘burn in’ and checked for a stationary phase of likelihood, checking for ESS values higher than 200 for all parameters on Tracer 1.7 [56]. The clades’ posterior probabilities (PP) were based on the majority rule consensus, using the stored trees, and calculated with MrBayes 3.1.2 [54]. Support values are presented below the branches, with posterior probabilities before bootstrap values.

### 4.4. Character-Mapping

Macro and micromorphological characters were scored for all species sampled in this study. Character coding followed the recommendations of [57] for morphological phylogenies. Primary homology hypotheses [58] were proposed for binary and multistate characters (Table 2). The author reviewed and compiled leaf anatomical characters and their corresponding character states with systematic value in Asteraceae. These data were obtained from a bibliographical review, focusing on studies published in journals and deposited in repositories. All characters were optimised on the concatenated tree using the Maximum Likelihood function mk1 using Mesquite 2.73 [59], visualised on Winclada [60], and edited in CorelDRAW 2020^®^ software.

### 4.5. Taxonomy

The terminology used in the taxonomy section follows Radford et al. [3,61]. Maps were elaborated using the ArcGIS 9.3 software [62], geographical coordinates were obtained from SpeciesLink [63], and shapefiles were obtained from WWF (2023) [64]. The conservation status was proposed following the recommendations of IUCN Red List Categories and Criteria [65] (IUCN 2023). GeoCAT [66] was used for calculating the Extent of Occurrence (EOO) and the Area of Occurrence (AOO).

## 5. Conclusions

We present the first comprehensive and empirical study to score, code, optimise, and test for secondary homologies in 102 structural macro and microanatomical characters recovered from our anatomical analyses of leaves of the *Catolesia* clade and outgroups (Asteraceae). We recovered relevant anatomical synapomorphies for all genera within this clade and outgroups. Importantly, our integrative approach also revealed a distinct morphological and phylogenetic lineage corresponding to the new monotypic genus *Nadia praeficta*, supported by a combination of phylogenetic and leaf anatomical characters that clearly separate it from *Disynaphia* s.s. and other members of the Catolesia clade. This robust list of characters allows the standardisation of descriptions of anatomical leaf traits in Eupatorieae (Asteraceae). Thus, leaf anatomical studies in this tribe represent a valuable taxonomic tool for studies based on the evolution of anatomical leaf characters in Asteraceae.

## Figures and Tables

**Table 1:** Matrix of macro and micromorphological characters coded for the sampled species in this study. The dash (-) indicates non-applicable characters, including characters associated with absent structures, such as the petiole in sessile species.

*Catolesia clade*	*Disynaphia ligulifolia*	*Disynaphia littoralis*	*Disynaphia multicrenulata*	*Disynaphia spatulatha*	*Acritopappus confertus*	*Bahianthus viscosus*	*Bejaranoa semistriata*	*Catolesia huperzioides*	*Catolesia mentiens*	*Catolesia monocephala*	*Disynaphia praeficta*	*Lapidia apicifolia*	*Morithamnus crassus*	*Morithamnus ganophyllus*
0	0	0	0	0	1	0	1	0	0	0	0	1	0	0
1	1	1	1	1	0	1	1	2	2	0	0	1	1	1
2	0	0	0	0	1	1	1	0	0	0	1	1	1	1
3	0	0	0	0	1	1	0	0	0	0	1	1	1	1
4	2	6	0	2	5	7	1	0	7	3	6	4	1	7
5	3	0	0	3	4	3	3	0	0	3	0	0	3	0
6	0	0	0	0	1	0	0	0	0	3	0	1	1	0
7	1	1	3	1	1	1	2	2	2	2	1	2	2	0
8	0	0	1	0	0	2	3	0	0	0	1	1	0	0
9	0	0	0	0	0	0	0	1	1	1	0	0	0	0
10	0	0	0	0	0	0	0	0	0	0	0	0	1	1
11	1	1	1	1	0	0	0	1	1	1	1	1	0	0
12	0	0	0	0	0	0	0	0	0	0	3	3	0	0
13	0	0	0	0	0	0	0	1	0	0	0	0	0	0
14	-	-	-	-	1	1	0	-	-	-	0	0	1	1
15	-	-	-	-	0/2/3/4	0/1/3/4	0/1/3/4	-	-	-	0/1/4	0/1/2/4	0/1/4	0/1/2/3/4
16	-	-	-	-	0	0	0	-	-	-	1	1	1	1
17	-	-	-	-	0	0	0	-	-	-	0	0	0/1	0
18	-	-	-	-	0	1	0	-	-	-	0/1	1	0	0
19	-	-	-	-	0	0	0	-	-	-	1	1	0/1	0/1
20	-	-	-	-	1	1	1/2	-	-	-	1	1	1	1
21	-	-	-	-	1	1	0	-	-	-	1	1	1	1
22	-	-	-	-	0	0	0/1	-	-	-	0	0/1	0/1	0/1
23	-	-	-	-	0	0	0/1	-	-	-	0	0/1	0	0
24	-	-	-	-	1	1	1	-	-	-	1	0/1	0	0
25	-	-	-	-	0/1	0	0/1	-	-	-	0	0/1	0/1	0/1
26	-	-	-	-	0	0	0	-	-	-	1	0/1	1	1
27	-	-	-	-	1	1	1	-	-	-	0/1	0/1	0	0
28	-	-	-	-	-	-	0	-	-	-	-	-	-	-
29	-	-	-	-	-	-	1/2/3/4/5	-	-	-	-	-	-	-
30	-	-	-	-	-	-	0	-	-	-	-	-	-	-
31	-	-	-	-	-	-	1	-	-	-	-	-	-	-
32	-	-	-	-	0	0	0	-	-	-	0	0	0	0
33	-	-	-	-	0/1	0/1	0/1	-	-	-	0/1	0	0/1	0/1
34	-	-	-	-	0/1	0	0	-	-	-	0	0	0/1	0/1
35	-	-	-	-	1	1	1	-	-	-	1	1	1	1
36	-	-	-	-	1	1	1	-	-	-	1	1	1	1
37	-	-	-	-	0/1	1	1	-	-	-	1	1	1	1
38	-	-	-	-	1	1	1	-	-	-	1	1	1	1
39	-	-	-	-	0	0	0	-	-	-	0	0	0	0
40	-	-	-	-	1	1	1	-	-	-	1	1	1	1
41	-	-	-	-	0	0	0	-	-	-	0	0	0	0
42	-	-	-	-	2/4/5/6	3/4/6	4/5	-	-	-	0	1/2	4/5	8/9
43	-	-	-	-	1	0	1	-	-	-	1	1	1	1
44	-	-	-	-	1	1	0/1	-	-	-	0	0	0	1
45	0	1	1	0	2	1	1	0	1	3	0	0	2	2
46	-	0	0	-	-	0/1	0	-	0	-	-	-	-	-
47	1/4	0/4	0/2/4	0/3/4	0/3/4	0/1/3/4	0/1/4	0/4	0/1/4	0/1/4	0/1/4	0/1/2/4	0/1/4	0/1/2/3/4
48	1	1	1	1	0	0	0	0	0	1	1	1	1	1
49	0	0	0	0	0	0	0	0	0	0	0	0	0	0
50	1	1	1	1	0	1	0	1	1	1	1	1	1	1
51	1	1	0	1	1	1	0	0	0	0	0	1	1	0
52	1	1	0/1	0/1	0	0	0	0	0/1	0	1	0	0/1	0/1
53	1	1	1	1	1	1	1	0/1	1	1	1	1	1	1
54	0/1	0/1	1	1	0	0	0/1	0	0	0	0	0	0	0
55	1	0	0	0	1	1	0	1	1	1	1	0	1	1
56	0/1	0/1	0/1	0/1	0	0	0/1	0	0	0	0	0/1	0/1	0
57	0/1	0/1	0/1	0/1	0	0	0/1	0	0	0	0	0/1	0	0
58	1	1	1	1	1	1	1	1	1	0/1	1	0/1	1	1
59	0/1	0	0/1	0/1	0/1	0	0/1	0	0	0	0	0/1	0/1	0/1
60	0/1	1	0/1	1	0/1	1	0/1	1	1	1	1	0/1	1	1
61	0/1	0	0/1	0/1	1	1	1	0	0	0	0	0/1	0	0
62	0	0/1	0/1	0/1	-	-	0	-	-	-	-	-	-	-
63	2/3	1/2	2/3	2/3	-	-	1/2/3/4/5	-	-	-	-	-	-	-
64	0	0	0/1	0/1	-	-	0	-	-	-	-	-	-	-
65	0/1	1	0/1	0/1	-	-	1	-	-	-	-	-	-	-
66	0	0	0	0	0	0	0	0	0	1	0	0	0	0
67	0	0	0	0	0	0	0	0	4	0	0	0	0	0
68	0	0	0	0	1	1	1	2	2	1	1	1	2	1
69	0	0	0	0	1	0	1	0	0	-	0	0	0	0
70	1	1	1	1	1	1	1	1	1	1	0/1	0	0/1	0/1
71	1	1	1	1	1	1	1	1	1	1	0	0	1	1
72	0	0	0	0	1	1	1	0	0	0	1	1	1	1
73	-	-	-	-	1/2	1	0	-	-	-	-	1/3	1/2	0/1
74	0	0	0	0	1	1	1	1	1	1	1	1	1	1
75	-	-	-	-	1	0/2/3	1	1/3	1/3	1	1	1	1	1
76	0	0	0	0	1	0	0	0	0	0	0	0	0	0
77	1	1	0	1	0	1	0	0	0	-	0	1	0	0
78	1	1	1	1	1	1	1	0	1	1	1	1	1	1
79	0	0	0	0	0	0	0	0	0	0	0	0	0	0
80	1	1	1	1	1	1	1	1	1	1	1	1	1	1
81	0	0	0	0	0	0	0	0	0	0	1	0	0	0
82	0/1	0	0	0	6/7	1/2	2	0	0	6/7	0/2	3/4	5/6	3/5
83	1	1	1	1	1	1	1	1	1	1	1	1	1	1
84	0	0	0	0	2	0	2	0	0	2	0	0	2	2
85	1/2	1/2	1/2	1/2	1/2	1/2	1/2	1/2	1/2	1/2	1/2	1/2	1/2	1/2
86	1	0	0	1	0	1	0	0	1	1	0	1	0	0
87	1	1	0	1	0	0	0	0	0	0	0	0	0	0
88	0	0	1	0	0	1	0	1	0	0	1	0	0	0
89	-	-	2	-	-	2	-	2	-	-	0	-	-	-
90	-	-	1	-	-	0/1	-	1	-	-	0/1	-	-	-
91	1	1	1	1	0	1	0	1	1	0	1	1	1	1
92	-	-	-	-	0	-	0	-	-	-	-	-	-	-
93	1	1	1	1	2	1	2	2	2	1/2	2	2	2	2
94	0	0	0	0	2	0	0	0	0	0	0	0	0	0
95	0/1	0/1	0/1	0/1	1	1/2	0	0	0/1	1/2	0/1	1/2	0/1	0/1
96	0/1	0/1	0/1	0	1/2	0/1/2	1/2	0/1	1/2	0/1	4/6	7/8	4/5	4/5
97	1	0	0	0	1	0	0	0	0	0	0	0	0	0
98	0	0	0	0	1	0	1	0	0	-	0	0	1	1
99	1	1	1	1	0	0	0	0	0	-	1	1	0	0
100	0	0	0	0	0	0	1	0	0	-	0	0	0	0
101	0	0	0	0	0	1	0	0	0	-	0	0	0	0
0 Leaves, phyllotaxy, type: spirally alternate (0); opposed decussate (1) 1 Leaves, disposition on the apex of branches: congested (0); lax (1); imbricate (2) 2 Leaves, petiole: absent (0); present (1) 3 Leaves, lamina, viscosity: absent (0); present (1) 4 Leaves, lamina, shape: lanceolate (0); obovate (1); spatulate (2); acicular (3); orbicular (4); elliptic (5); linear (6); oblanceolate (7) 5 Leaves, lamina, apex: acute (0); apiculate (1); rounded (2); obtuse (3); caudate (4) 6 Leaves: straight (0); conduplicate (1); cylindrical (3) 7 Leaves, lamina, base: obtuse (0); attenuate (1); cuneate to truncate (2); rounded (5) 8 Leaves, lamina, margin: entire (0); dentate (1); serrate (2); crenate (3) 9 Leaves, lamina, consistency: membranous or coriaceous (0); fleshy or succulent (1) 10 Leaves, marcescent: absent (0); present (1) 11 Leaves, lamina, indument: conspicuous (0); inconspicuous (1) 12 Leaves, lamina, venation pattern: pinnate brochidodromous (0); pinnate- cladodromous (1); acrodromous (2); actinodromous (3); pinnate craspedodromous (4) 13 Leaves, lamina, venation pattern, intramarginal veins: absent (0); present (1) 14 Leaves, petiole, contour, shape: plane-convex (0); concave-convex (1) 15 Leaves, petiole, epidermal cells, transversal section, contour, type: squared (0); periclinally elongate (1); anticlinally elongate (2); rounded (3); irregularly polygonal (4) 16 Leaves, petiole, epidermis, cell walls, anticlinal surfaces, contour: straight (0); sinuous (1) 17 Leaves, petiole, epidermis, layers, number: uniseriate (0); inconspicuous biseriate (1) 18 Leaves, petiole, epidermis, cuticle, thickness: thick (0); delgate (1) 19 Leaves, petiole, epidermis, cuticle, texture: striate (0); smooth (1) 20 Leaves, petiole, trichomes, presence/type: absent (0), glandular (1); tector (2) 21 Leaves, petiole, trichome, epidermis, midrib, density: dense (0); lax (1) 22 Leaves, petiole, glandular trichomes, layers, number: uniseriate (0); biseriate (1) 23 Leaves, petiole, epidermis, glandular trichomes, peduncle, layers, number: uniseriate (0); biseriate (1); multiseriate (2) 24 Leaves, petiole, epidermis, glandular trichome, head shape: ellipsoid (0); globoid (1) 25 Leaves, petiole, epidermis, glandular trichomes, head: uniseriate (0); biseriate (1) 26 Leaves, petiole, epidermis, glandular trichomes, head, lobes: bilobed (0); not lobed (1) 27 Leaves, petiole, epidermis, glandular trichome, insertion: in a depression (0); same level as epidermal cells (1) 28 Leaves, petiole, epidermis, tector trichome, shape: conical (0); cylindrical (1) 29 Leaves, petiole, epidermis, tector trichomes, cell number: one (0); two (1); three (2); four (3); five (4); eight (5) 30 Leaves, petiole, epidermis, tector trichomes, base cells, number: one (0); two (1) 31 Leaves, petiole, epidermis, tector trichome, insertion: in a depression (0); same level as epidermal cells (1) 32 Leaves, petiole, epidermis, trichomes, cluster disposition: absent (0); present (1) 33 Leaves, petiole, epidermis, stomata, position in relation to epidermal cells: raised (0); plane (1) 34 Leaves, petiole, collenchyma, layers, number: three (0); four (1), five (2) 35 Leaves, petiole, sclerenchyma, presence: absent (0); present (1) 36 Leaves, petiole, fibres, distribution: midrib (0); vascular bundles (1) 37 Leaves, petiole, chlorophyll parenchyma, presence: absent (0); present (1) 38 Leaves, petiole, chlorophyll parenchyma, type: palisade (0); spongy (1) 39 Leaves, petiole: secretory cavity, presence: absent (0); present (1) 40 Leaves, petiole, ducts, presence: absent (0); present (1) 41 Leaves, petiole, secretory ducts, distribution: only near bundles (0); all petiole (1) 42 Leaves, petiole, vascular bundles, number: three (0); four (1); five (2); six (3); seven (4); eight (5); nine (6); ten (7); eleven (8); twelve (9) 43 Leaves, petiole, accessory bundles: absent (0); present (1) 44 Leaves, petiole, vascular bundles, arrangement: horizontal (0); U-shaped (1); V-shaped (2) 45 Leaves, lamina, midrib, contour: convex-convex (0); plane-convex (1); concave-convex (2); sub unifacial (3) 46 Leaves, lamina, midrib, plane-convex contour, adaxial surface, shape: slightly projected (vertical elevation on the wall) (0); central depression (1) 47 Leaves, lamina, epidermal cells, transversal section, contour, type: squared (0); periclinally elongate (1); anticlinally elongate (2); rounded (3); irregularly polygonal (4) 48 Leaves, lamina, epidermis, cell walls, anticlinal surfaces, contour: straight (0); sinuous (1) 49 Leaves, epidermis, layers, number: uniseriate (0); inconspicuous biseriate (1) 50 Leaves, lamina, epidermal cells, volume difference on abaxial and adaxial surfaces: larger cells on adaxial surface and smaller cells on abaxial surface (0); cells of similar volume on both surfaces (1) 51 Leaves, lamina, epidermis, cuticle, thickness: thick—1/3 of the cell wall height (0); delgate—lower than 1/3 of the cell wall height (1) 52 Leaves, lamina, epidermis, cuticle, texture: striate (0); smooth (1) 53 Leaves, lamina, epidermis, trichomes, presence: absent (0); present (1) 54 Leaves, lamina, epidermis, trichomes, type: glandular (0); glandular and tector (1) 55 Leaves, lamina, epidermis, midrib, density: dense (0); lax (1) 56 Leaves, lamina, epidermis, glandular trichomes, number: uniseriate (0); biseriate (1) 57 Leaves, lamina, epidermis, glandular trichomes, peduncle, layers, number: uniseriate (0); biseriate (1); multiseriate (2) 58 Leaves, lamina, epidermis, glandular trichome, head shape: ellipsoid (0); globoid (1) 59 Leaves, lamina, epidermis, glandular trichomes, number head: uniseriate (0); biseriate (1) 60 Leaves, lamina, epidermis, glandular trichomes, head, lobes: bilobed (0); not lobed (1) 61 Leaves, lamina, epidermis, glandular trichome, insertion: in a depression (0); same level as epidermal cells (1) 62 Leaves, lamina, epidermis, tector trichome, shape: conical (0); cylindrical 63 Leaves, lamina, epidermis, tector trichomes, cell number: one (0); two (1); three (2); four (3); five (4); eight (5) 64 Leaves, lamina, epidermis, trichomes, base cells, number: one (0); two (1) 65 Leaves, lamina, epidermis, tector trichome, insertion: in a depression (0); same level as epidermal cells (1) 66 Leaves, lamina, epidermis, trichomes, cluster disposition: absent (0); present (1) 67 Leaves, lamina, epidermis, trichomes, distribution: all lamina (0); only on midrib (1); only on semi limb (2); only on apex (3); only on margins (4) 68 Leaves, lamina, epidermis, stomata, type: anisocytic (0); anomocytic (1); actinocytic (2); diacytic (3); tetracytic (4); paracytic (5); tetracytic (6) 69 Leaves, lamina, epidermis, stomata, distribution: amphistomatic (0); hypostomatic (1); spystomatic (2) 70 Leaves, lamina, epidermis, stomata, position in relation to epidermal cells: raised (0); plane (1) 71 Leaves, laminae, epidermis, stomata, subsidiary cells, cell wall, external periclinal surface, striae, presence: absent (0); present (1) 72 Leaves, lamina, collenchyma, presence: absent (0); present (1) 73 Leaves, lamina, collenchyma, layer, number: two (0); three (1); four (2); seven (3) 74 Leaves, lamina, sclerenchyma, presence: absent (0); present (1) 75 Leaf, lamina, fibres, distribution: midrib (0); vascular bundles (1); mesophyll (2); margin (3) 76 Leaves, lamina, hypodermis, presence: absent (0); present (1) 77 Leaves, lamina, midrib, proportion in relation to mesophyll: more voluminous than mesophyll (0); as voluminous as mesophyll (1) 78 Leaves, lamina, midrib, chlorophyll parenchyma, presence: absent (0); present (1) 79 Leaves, lamina, secretory cavity, presence: absent (0); present (1) 80 Leaves, lamina, ducts, presence: absent (0); present (1) 81 Leaves, lamina, secretory ducts, distribution: only near bundles (0); all over lamina (1) 82 Leaves, lamina, midrib, vascular bundles, number: one (0); two (1); three (2); four (3); five (4); seven (5); eight (6); ten (7); eleven (8); twelve (9) 83 Leaves, lamina, vascular bundles, arrangement: irregular (0); regular (1) 84 Leaves, lamina, vascular bundles, regular arrangement, type: horizontally disposed to midrib (0); U-shaped (1); V-shaped (2) 85 Leaves, lamina, epidermis, chemical nature: phenolic compounds (0); oils (1); pectin (2); phloroglucinol-HCl (3) 86 Leaves, lamina, oil bodies, presence: absent (0); present (1) 87 Leaves, lamina, Kranz anatomy, presence: absent (0); present (1) 88 Leaves, lamina, sheath extension, presence: absent (0); present (1) 89 Leaves, lamina, sheath cells extension, type: collenchymal (0); sclerenchymal (1); parenchymal (2) 90 Leaves, lamina, sheath, distribution: chlorophyll parenchyma (0); veins (1) 91 Leaves, lamina, mesophyll organisation: dorsiventral (0); isobilateral (1) 92 Leaves, lamina, mesophyll, palisade parenchyma extension: up to margins (0); ending before margins (1) 93 Leaves, lamina, palisade parenchyma, shape: anticlinally rectangular elongate cells (0); long cylindrical (1); short cylindrical (2) 94 Leaves, lamina, spongy parenchyma, cell, shape: isodiametric (0); orbicular (1); oval (2); polygonal (3) 95 Leaves, lamina, palisade parenchyma, layers, number: one (0); two (0) 96 Leaves, lamina, spongy parenchyma, layers, number: three (0); four (1); five (2); seven (3); eight (4); nine (5); ten (6); eleven (7); twelve (8) 97 Leaves, lamina, plicate parenchyma, presence: absent (0); present (1) 98 Leaves, lamina, semi-limb angle towards midrib: equalling 90° (0); lower than 90° (1) 99 Leaves, lamina, semi limb, undulation, presence: absent (0); present (1) 100 Leaves, lamina, semi-limb margin, shape: straight (0); revolute (1) 101 Leaves, lamina, margin with cholenchymatic extension: absent (0); present (1)														

**Table 2:** Homoplasies and apomorphies (in bold) recovered for all studied lineages according to the Winclada tree.

Lineages	Characters (States) Recovered as Homoplasies (H) or Apomorphies (S). X = No Morphological Characters
Clade A	X
Clade B (*Disynaphia* S.S.)	X
Clade C	**S: 87(1)**
Clade D	X
Clade E	X
Clade F	**S: 11(0); 48(0); 61(1)**
*Acritopappus*	H: 0(1); 1(0); 6(1); 50(0); 69(1) 84(2); 91(0); **S:4(5); 76 (1); 94(2)**
*Bejaranoa*	H: 88(1); **S: 8(2); 43(0); 75(0,2,3); 101(1)**
Clade G	**S:7(2)**
*Lapidia*	H: 0(1); 6(1); 8(1); 12(3); 71(0); **S: 4(4); 42(1,2); 70(0); 82(3,4)**
Clade H	X
Clade I (*Morithamnus*)	H: 11(0); 45(2); 98(1) **S: 10(1); 24(0); 27(0); 82(5)**
Clade J	X
*Disynaphia praeficta*	H: 4(6); 7(1); 8(1); 12(3); 52(1); 71(0); 88(1); **S: 42(0); 81(1); 89(0)**
Clade K	H: 3(0)
*Bahianthus*	H: 0(1); 4(1); 11(0); 50(0); 55(0); 61(1); 69(1); 98(1); **S: 8(3); 100(1)**
Clade L (*Catolesia*)	H: 2(0); 72(0); **S: 9(1)**
Clade M	H:68(2)

**Table 3:** Species of the *Catolesia* clade and outgroups anatomically sampled in this study, followed by their vouchers, localities, and herbarium accession numbers. (*) Field-collected specimens. (**) Herbarium collected specimens.

Species	Populations/Collector and Number	Localities	Voucher
*Bahianthus viscosus* (Spreng.) R.M. King & H. Rob. *	Guterres, A.V.F. & et al. 384	Mucugê, Bahia, Brazil	ALCB137862
	Guterres, A.V.F. & et al. 401	Mucugê, Bahia, Brazil	BHCB205938
	Guterres, A.V.F. & et al. 402	Mucugê, Bahia, Brazil	BHCB205967
*Catolesia huperzioides* Roque, H. Rob. & A.A. Conceição	Amorim, V.O. 410	Palmeiras, Bahia, Brazil	ALCB133476
	Funch, R. 677	Mucugê, Bahia, Brazil	HUEFS123167
	Conceição, A.A. 3221	Mucugê, Bahia, Brazil	HUEFS141602
*Catolesia mentiens* D.J.N. Hind	Guedes et al. 12,745	Abaíra, Bahia, Brazil	ALCB 75256
	Amorim, V.O.; Coelho, J. 440	Abaíra, Bahia, Brazil	ALCB 133475
	Ferreira, S.C. 360	Abaíra, Bahia, Brazil	HUEFS 142816
*Catolesia monocephala* Roque & S.C. Ferreira	Amorim, VO 408	Palmeiras, Bahia, Brazil	ALCB133478
	Amorim, VO 411	Palmeiras, Bahia, Brazil	ALCB133475
	Amorim, VO; Gandara, A 521	Palmeiras, Bahia, Brazil	ALCB133477
*Disynaphia praeficta* (B.L. Rob.) R.M. King & H. Rob.	Roque, N. & et al. 4718	Gouveia, Minas Gerais, Brazil	ALCB120892
	Roque, N. & et al. 5224	Diamantina, Minas Gerais, Brazil	ALCB138392
	Hattori, E.K.O. & et al. 939	Itacambira, Minas Gerais, Brazil	BHCB134221
	Mota, N.F.O. & et al. 1801	Diamantina, Minas Gerais, Brazil	BHCB144478
*Lapidia apicifolia* Roque & S.C. Ferreira *	Staudt, M.G. & Barres, L. 118	Morro do Chapéu, Bahia, Brazil	ALCB75951
	Guterres, A.V.F. & et al. 376	Morro do Chapéu, Bahia, Brazil	ALCB135976
	Gandara, A. & Amorim, V.O. 138	Morro do Chapéu, Bahia, Brazil	ALCB129268
	Roque, N. & Alunos de Botânica III 4514	Morro do Chapéu, Bahia, Brazil	ALCB117425
*Morithamnus crassus* R.M. King et al. *	Guterres, A.V.F. & et al. 385	Mucugê, Bahia, Brazil	ALCB137863
	Guterres, A.V.F. & et al. 386	Mucugê, Bahia, Brazil	ALCB137864
	Guterres, A.V.F. & et al. 403	Mucugê, Bahia, Brazil	BHCB205968
*Morithamnus ganophyllus* (Mattf.) R.M. King & H. Rob. *	Guterres, A.V.F. & et al. 405	Piatã, Bahia, Brazil	BHCB205970
	Guterres, A.V.F. & et al. 404	Piatã, Bahia, Brazil	BHCB205969
	Guterres, A.V.F. & et al. 388	Piatã, Bahia, Brazil	ALCB137866
*Acritopappus confertus* (Gardner) R.M. King & H. Rob.*	Guterres, A.V.F. & et al. 378	Morro do Chapéu, Bahia, Brazil	ALCB135977
	Guterres, A.V.F. & et al. 382	Mucugê, Bahia, Brazil	ALCB137861
	Guterres, A.V.F. & et al. 381	Morro do Chapéu, Bahia, Brazil	BHCB205965
*Bejaranoa semistriata* (Baker) R.M. King & H. Rob.	Gandara, A. & et al. 22	Licínio de Almeida, Bahia, Brazil	ALCB120813
	Bautista, H.P. & Rodriguez-Oubiña, J. 2404	Antônio Gonçalves, Bahia, Brazil	ALCB127971
	Melo, E. & et al. 12,058	Morro do Chapéu, Bahia, Brazil	ALCB118865
*Disynaphia multicrenulata* (Baker) R.M. King & H. Rob.**	Pivetta, J. 383	Augusto Pestana, Rio Grande do Sul, Brazil	BHCB166490
*Disynaphia ligulifolia* (Hook. & Arn.) R.M. King & H. Rob.**	Hattori, E.K.O. & et al. 1110	Cambará, Rio Grande do Sul, Brazil	BHCB137532
	Hattori, E.K.O. & et al. 1140	Bom Jesus, Rio Grande do Sul, Brazil	BHCB137562
	Hattori, E.K.O. & et al. 1099	Cambará do Sul, Rio Grande do Sul, Brazil	BHCB137521
*Disynaphia littoralis* (Cabrera) R.M. King & H. Rob.**	Hattori, E.K.O. & et al. 1334	Campina Grande do Sul, Paraná, Brazil	BHCB159325
	Hattori, E.K.O. & et al. 1126	Painel, Santa Catarina, Brazil	BHCB137548
	Fernandes, A.C. 979	Ponta Grossa, Paraná, Brazil	BHCB162717
*Disynaphia spathulata* (Hook. & Arn.) R.M. King & H. Rob.**	Gotijo, F.D. 681	Caeté, Minas Gerais, Brazil	BHCB178056
	Hattori, E.K.O. & et al. 1170	Distrito Federal, Brasilia, Brazil	BHCB137464
	Rezende, S.G. 1335	Nova Lima, Minas Gerais, Brazil	BHCB106112

**Table 4:** GenBank accession numbers of all species sampled in this study.

Species	ETS	ITS	*ndhI*	*ndhF*
*Disynaphia ligulifolia*	KP454653.1	KP454348.1	-	-
*Disynaphia littoralis*	KP454654.1	-	-	-
*Disynaphia multicrenulata*	KP454655.1	KP454349.1	KP454207.1	KP454927.1
*Disynaphia spathulata*	KP454657.1	-	KP454209.1	KP454929.1
*Acritopappus confertus*	KP454597.1	KP901188.1	KP454162.1	KP454885.1
*Bejaranoa semistriata*	KP454625.1	KP901154.1	KP454186.1	KP454907.1
*Bahianthus viscosus*	KP454619.1	KP901152.1	P454180.1	KP454902.1
*Catolesia huperzioides*	KP454633.1	KP901159.1	KP454196.1	-
*Catolesia mentiens*	KP454634.1	KP901158.1	-	KP454917.1
*Catolesia monocephala*	-	KP901168	-	-
*Disynaphia praeficta*	KP454656.1	KP454350.1	KP454208.1	KP454928.1
*Lapidia apicifolia*	-	KP901164	-	-
*Morithamnus crassus*	KP454701.1	KP901169.1	KP454249.1	KP454966.1
*Morithamnus ganophyllus*	KP454702.1	KP901170.1	-	-

## Data Availability

The data supporting the findings of this study are available within the article.
